# DctR contributes to the virulence of avian pathogenic *Escherichia coli* through regulation of type III secretion system 2 expression

**DOI:** 10.1186/s13567-021-00970-6

**Published:** 2021-07-06

**Authors:** Yaodong Zhang, Yao Wang, Hong Zhu, Zhengfei Yi, Dossêh Jean Apôtre Afayibo, Chenglin Tao, Tao Li, Mingxing Tian, Jingjing Qi, Chan Ding, Shengqing Yu, Shaohui Wang

**Affiliations:** Shanghai Veterinary Research Institute, Chinese Academy of Agricultural Sciences, Shanghai, 200241 China

**Keywords:** Avian pathogenic *E. coli*, DctR, virulence, *E*. *coli* type III secretion system 2, regulation

## Abstract

Pathogens could precisely alter their gene expression to facilitate their survival and successful infection. The LuxR family transcriptional regulator DctR (also known as YhiF) was shown to participate in the regulation of acid fitness and adhesion of enterohemorrhagic *E*. *coli* (EHEC) O157:H7. Avian pathogenic *Escherichia coli* (APEC) causes significant economic losses to the poultry industries and also potentially threatens human health. However, the effects of DctR on the fitness and virulence of APEC have not been investigated yet. To assess the function of DctR in APEC, the *dctR* gene mutant and complemented strains were constructed and biologically characterized. Our results show that inactivation of the *dctR* gene led to decreased biofilm formation, diminished serum resistance, reduced adherence capacity, attenuated colonization and virulence of APEC in ducks. The altered capacities of the mutant strain were restored by genetic complementation. In addition, we found that DctR positively regulates the expression of *E*. *coli* type III secretion system 2 (ETT2) core genes in APEC. The expression of the inflammatory cytokines interleukin (IL)-1β and IL-8 were decreased in HD-11 macrophages infected with the mutant strain compared with the wild-type strain. These observations indicate that regulator DctR contributes to the virulence of APEC through regulation of ETT2 expression.

**Supplementary Information:**

The online version contains supplementary material available at 10.1186/s13567-021-00970-6.

## Introduction

Pathogenic *Escherichia coli* (*E*. *coli*) are remarkably diverse, and they cause a wide range of diseases, ranging from gastroenteritis and diarrhea to extraintestinal infections, such as neonatal meningitis, urinary tract infections, pneumonia and septicemia [1, 2]. Avian pathogenic *E*. *coli* (APEC), belonging to a subset of extraintestinal pathogenic *E*. *coli* (ExPEC), could cause aerosacculitis, polyserositis, septicemia and other extraintestinal diseases in chickens, turkeys and other avian species [3]. The colibacillosis caused by APEC is a significant economic burden on the poultry industries worldwide [4]. In addition, APEC shares significant genetic similarities and a similar pathogenic mechanism with human ExPEC, including uropathogenic *E. coli* (UPEC) and neonatal meningitis *E. coli* (NMEC) [5–7]. Thus, APEC potentially serve as a reservoir of virulence and resistance genes. In recent years, due to economic losses, drug resistance, and zoonotic potential, APEC has attracted heightened awareness [8]. APEC initially infects poultry via the respiratory tract, then spreads systemically through the bloodstream and cause multi-systemic infections. Although various virulence factors contributing to APEC pathogenesis have been identified [4], little is known of the virulence regulation of APEC.

Pathogenic bacteria are able to sense the environmental cues and alter its gene expression in a rapidly and effective manner to facilitate their survival and successful infection. Various regulatory mechanisms and complex regulatory network exist in *E*. *coli* that enhance bacterial fitness under different environmental conditions [9–11]. Among them, transcriptional regulators can activate or repress gene expression. LuxR family transcriptional regulators are present in a variety of bacteria, which harbor helix-turn-helix (HTH) DNA binding domain for transcriptional regulation. Various members of LuxR family transcriptional regulators, such as RcsA, RcsB, MatA, BglJ, YjjQ, have been characterized and are implicated in regulation of virulence, metabolism, antibiotic biosynthesis, motility, plasmid transfer, bioluminescence and biofilm formation [12–16]. The *dctR* (also known as *yhiF*) gene located in the acid fitness island of *E*. *coli*, encodes a LuxR family transcriptional regulator [17]. It has been demonstrated that DctR participates in the regulation of acid metabolite gene C4 transporter *dctA* and provides acid fitness for *E*. *coli* [18, 19]. In addition, DctR also acts as a negative regulator for the expression and function of enterocyte effacement (LEE) type III secretion system (T3SS) locus in enterohemorrhagic *E*. *coli* (EHEC) O157:H7 [20–22]. RcsB, the response regulator of Rcs two-component signal transduction system (TCS), could regulate the transcription either as a homodimer or together with auxiliary LuxR regulators, including DctR [23]. However, the function, regulon and regulatory mechanism of DctR in pathogenic *E*. *coli* remains unclear.

In this study, we attempted to determine the effects of the LuxR family transcriptional regulator DctR on the phenotype and pathogenicity of APEC. The results indicate that DctR was required for biofilm formation, adhesion, survival in serum, colonization and virulence of APEC. Moreover, we found DctR positively regulates the expression of *E*. *coli* type III secretion system 2 (ETT2), which may contribute to the survival fitness and pathogenicity of APEC.

## Materials and methods

### Bacterial strains, plasmids and growth conditions

The bacterial strains and plasmids used in this study are listed in Table 1. The APEC wild-type strain CE08 (O2: K1) was originally isolated from the brain of a chicken clinically diagnosed with colisepticemia, which belonged to the phylogenetic *E*. *coli* reference (ECOR) group B2. It has been confirmed that CE08 could cause severe colibacillosis symptoms, including meningitis in the neonate and high mortality in ducks, chickens and mice model. All *E. coli* strains were grown in Luria–Bertani (LB) medium at 37 °C with aeration. When necessary, the antibiotics ampicillin (Amp, 100 μg/mL) or chloramphenicol (Cm, 35 μg/mL) were supplemented into the medium.

**Table 1:** **Bacterial strains and plasmids.**

Strains or plasmids	Characteristics	References
Strains		
CE08	APEC wild-type strain, O2: K1 serotype	This study
ΔdctR	*dctR* deletion mutant in CE08	This study
CΔdctR	ΔdctR with plasmid pSTV28-dctR	This study
DH5α	F-, *Δ(lacZYA-argF)U169, recA1, endA1, hsdR17(rk-, mk* +*), phoA, supE44, λ-*	TIANGEN
Plasmids		
pSTV28-P_*amp*_	Cm, lacZ, P_*amp*_ promoter	This study
pSTV28-dctR	pSTV28-P_*amp*_ derivative harboring *dctR* gene	This study
pKD46	Amp; expresses λ red recombinase	[24]
pKD3	Cm, template plasmid	[24]
pCP20	Cm, Amp, yeast Flp recombinase gene, FLP	[24]

### Construction of mutant and complemented strains

The *dctR* gene deletion mutant strain was generated using the lambda Red recombinase system [24] with some modifications and appropriate primers (Additional file 1). Briefly, linear PCR products with chloramphenicol resistance cassettes flanked by *dctR* upstream and downstream sequences were transformed into CE08 containing pKD46 to replace the *dctR* gene. Then, the chloramphenicol resistance cassette was eliminated by the plasmid pCP20. For complemented strain construction, the *dctR* gene was amplified and inserted into plasmid pSTV28-P_*amp*_. The recombinant plasmid pSTV28-dctR was transformed into mutant strain ΔdctR to generate complemented strains CΔdctR. The obtained mutant and complemented strains were validated by PCR and sequencing.

### Growth curve and motility assays

The growth kinetics and motility haloes of CE08, ΔdctR and CΔdctR strains were determined in LB medium and plates as described previously [25]. Briefly, the bacteria were incubated at 37 °C with shaking, and the growth of each strain was monitored for optical density 600 nm (OD_600nm_) value at 1 h interval. Bacterial motility haloes on LB soft agar motility plates (0.5% agar) were measured after incubation at 37 °C for 12 h.

### Serum bactericidal assay

Bacterial resistance to serum bactericidal was performed as described previously with some modifications [26]. Briefly, bacteria were incubated with various dilutions (12.5%, 25%, 50% and 100%) of SPF chicken serum at 37 °C for 30 min. Heat-inactivated serum was used as a negative control. The surviving bacteria were enumerated by plating on LB plates.

### Biofilm formation assays

The determination of biofilm formation of APEC strains was performed via quantitative analysis as described previously with minor modification [27, 28]. Briefly, the fresh bacterial culture was normalized as the OD_600nm_ of 1, which was diluted into LB supplemented with 0.5% glucose (W/V). Aliquots of 200 µL for each strain were dispensed in a 96-well plate and cultured without shaking at 37 °C for 24 h. Negative control contained uninoculated LB medium. Then, the planktonic bacterial cells were removed and the biofilms were stained with 200 μL crystal violet (0.1%, W/V) for 30 min. After rinsing and drying, the biofilms were solubilized and measured at OD_595nm_.

### Bacterial adhesion and invasion assays

The bacterial adhesion and invasive capacities were assessed as described previously [26] using chicken embryo fibroblast DF-1 cells. In brief, the DF-1 monolayers were washed with Dulbecco modified Eagle medium (DMEM) without fetal bovine serum (FBS) and infected with bacteria at a multiplicity of infection (MOI) of 100 for 2 h at 37 °C in CO_2_ incubator. After washing, infected cells were lysed with 0.5% Triton X-100 and the bacteria adhesion onto the cells were counted via plating on LB plates. For invasion assays, the APEC infected DF-1 cells were treated with DMEM containing gentamicin (100 μg/mL) for 1 h to kill extracellular bacteria. Then, the cells were washed, lysed and the invasive bacteria were enumerated by plating on LB plates. *E*. *coli* K12 MG1655 was used as the negative control.

### Impact of DctR on the virulence and systemic infection of CE08 in vivo

The effect of *dctR* on APEC virulence was investigated in duck models as previously described [29, 30]. Groups of ten 7-day-old ducks were challenged intramuscularly with bacteria at 10^6^ colony-forming units (CFUs) /duck. The virulence test was also performed by intratracheal infection. Ducks inoculated with PBS were used as negative controls. The survival/mortality was monitored daily until 7 days after infection.

To determine the effect of *dctR* on APEC colonization in vivo, the systemic infection experiment of the duck model was conducted as described previously [29–31]. Briefly, ducks were infected intramuscularly or intratracheally with bacterial suspension containing 10^8^ CFUs and euthanized humanely at 24 h post-infection. The liver, spleen and blood were collected, weighed and homogenized. Serial dilutions of the homogenates were plated onto LB plates to count the bacterial numbers. The colonization and proliferative capacity in the liver, spleen and blood of these APEC strains were compared.

### RNA isolation and quantitative real-time PCR (qRT-PCR)

The regulation roles of *dctR* on the ETT2 and virulence gene expressions were investigated by qRT-PCR as described previously [25]. In addition, the mRNA levels of cellular inflammatory cytokines in HD-11 cells infected with APEC strains were also measured via qRT-PCR [26]. In brief, total RNA was isolated from bacterial cultures (logarithmic phase) or bacteria-infected HD-11 cells using TRIzol® reagent (Invitrogen, Carlsbad, CA, USA). The contamination DNA removal and cDNA synthesis were performed using Turbo DNA-free kit (ThermoFisher) and PrimeScript® RT reagent kit (TaKaRa) according to the manufacturer's intstructions. qRT-PCR was performed to analyze the transcription levels of ETT2 genes and inflammatory cytokines using SYBR®Premix Ex Taq™ (TaKaRa). The relative gene transcription was normalized to the housekeeping gene *dnaE* (APEC) or *β-actin* (HD-11 cells) via the ΔΔCt method [32].

### Statistical analyses

Statistical analyses were conducted using the GraphPad Software package (GraphPad Software, La Jolla, CA, USA). One-way analysis of variance (ANOVA) and two-way ANOVA were used to analyze biofilm formation, bacterial adherence, invasion data and bactericidal serum, and qRT-PCR results, respectively. The comparison of the bacterial loads in the animal infection study were evaluated by the nonparametric Mann–Whitney U Test. The mean values are shown in the figures. Statistical significance was considered at *P* < 0.05.

## Results

### Inactivation of *dctR* has no effect on the growth and motility of CE08

**Figure 1:**
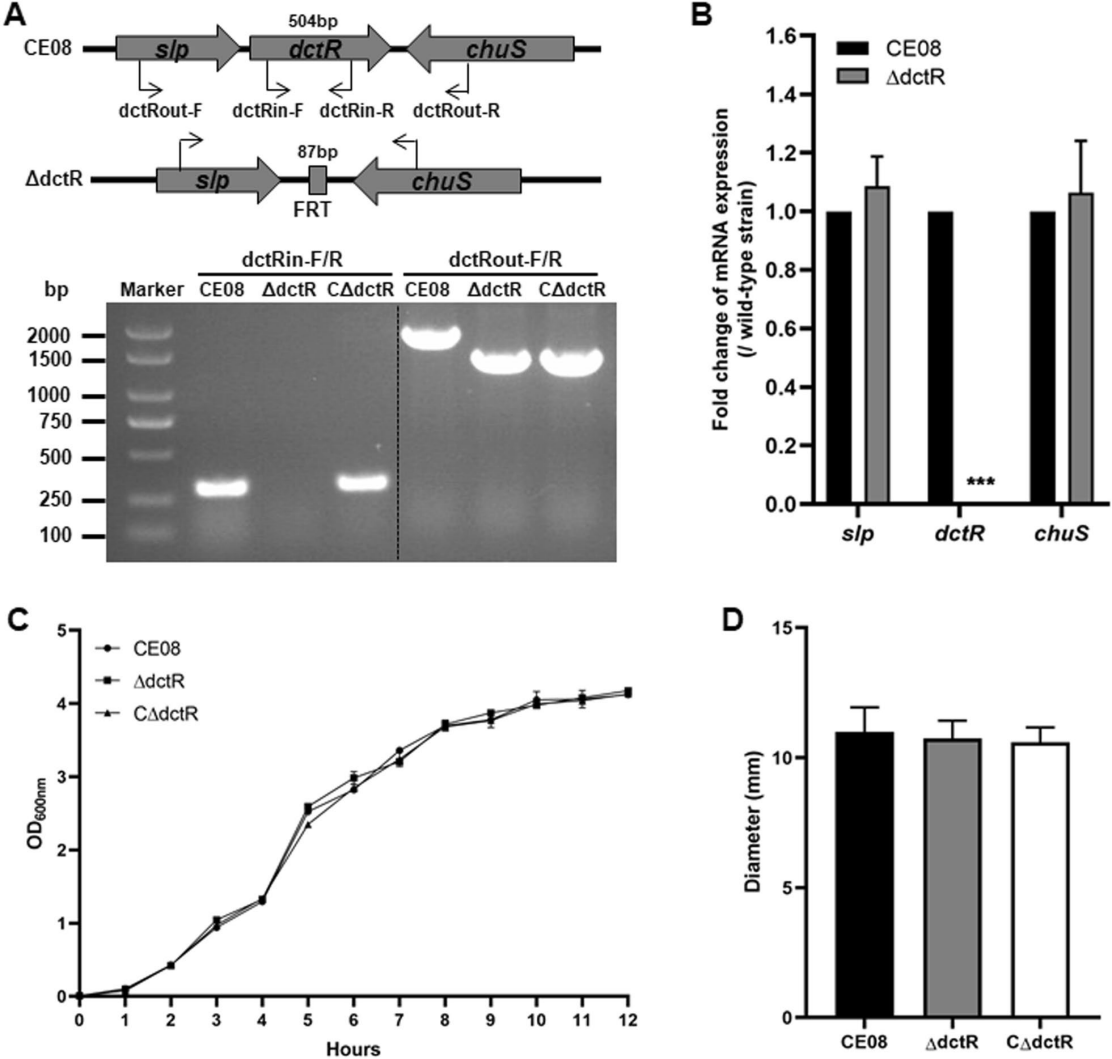
**Identification and characteristics of the *dctR* gene mutant and complemented strains**. **A** Schematic chart and confirmation of the *dctR* gene mutant and complemented strains by PCR. The *dctR* gene was replaced by the FRT site in the mutant strain. The primers used for the PCR identification of the *dctR* gene deletion are also indicated. The amplification of the corresponding target genes indicate the success in mutant and complemented strain construction. **B** Transcriptional level analysis of *dctR* and its flanking genes by qRT-PCR. Deletion of *dctR* did not affect the expression of the flanking genes *slp* and *chuS*. Moreover, no transcription of *dctR* was observed in the mutant strain. **C** Bacterial growth curves. APEC strains were grown in LB at 37 °C, and the OD_600nm_ was measured every hour. Mutant strain ΔdctR shows similar growth rate with the wild-type and complemented strains. **D** Bacterial motility. The motility diameter of the mutant strain was similar with those of the wild-type and complemented strains. Each value represents the average of three independent experiments. Significant differences were detected using one-way ANOVA (***P < 0.001).

The *dctR* gene mutant and complemented strains of APEC isolate CE08 were constructed and confirmed by PCR amplification using the respective primers. The PCR and sequencing validated the successful construction of mutant and complemented strains (Figure 1A). To determine whether *dctR* gene deletion had a polar effect, the transcription levels of *dctR* and its flanking genes *slp* and *chuS* in the wild-type and mutant strains were analyzed by qRT-PCR. The results show that the *dctR* gene was not transcribed in the mutant strain, and the transcriptions of the upstream gene *slp* and downstream gene *chuS* were not affected by *dctR* deletion (Figure  1B). These data suggest that *dctR* gene mutant had no polar effect. The growth curve shows that mutant strain ΔdctR grew similarly with the wild-type and complemented strains (Figure  1C). Moreover, no significant difference of the halo diameter on swarming agar plates was observed among these APEC strains (Figure  1D). These data indicate that deletion of *dctR* had no obvious effect on the growth and motility of CE08.

### *dctR* contributes to biofilm formation of CE08

**Figure 2:**
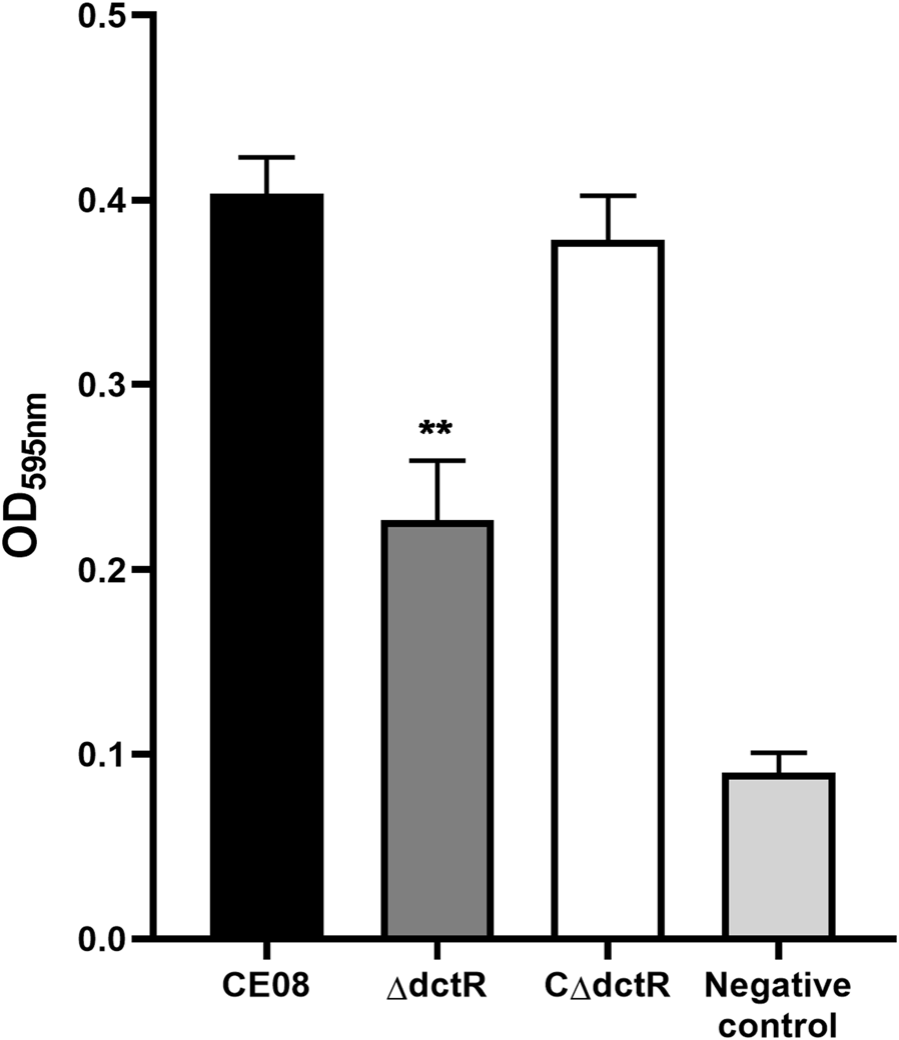
**Determination of biofilm formation by APEC strains**. Biofilm formation in 96-well plates was measured by crystal violet staining at OD_595nm_. The biofilm formation of the mutant strain ΔdctR was reduced by 43.8% as compared with the wild-type strain. Statistical significance was determined using one-way ANOVA (**P < 0.01).

To determine whether *dctR* was involved in biofilm formation, we measured and compared the biofilm formation capacity of wild-type, mutant and complemented strains. The inactivation of *dctR* results in a significantly decreased biofilm formation capacity compared with the wild-type strain CE08 (*P* < 0.01). Moreover, the complemented strain restored biofilm formation capacity to the level of the wild-type strain (Figure  2). These results indicate that DctR promoted the biofilm formation of CE08.

### *dctR* facilitates the serum resistance of CE08

**Figure 3:**
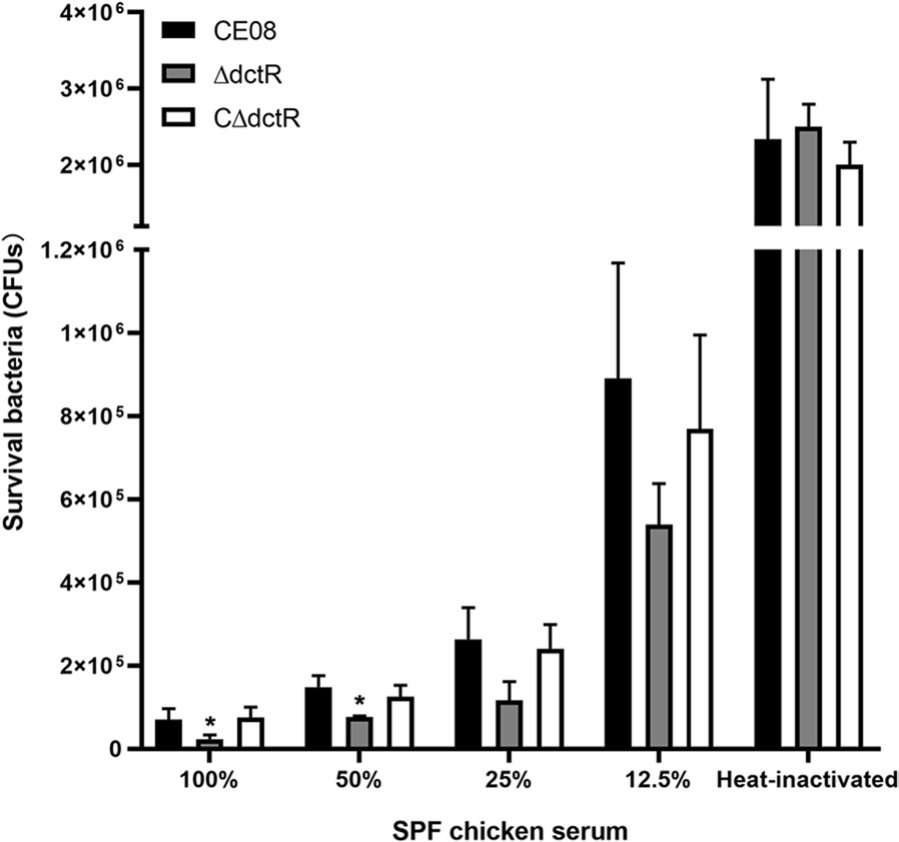
**Bacterial resistance to specific-pathogen-free (SPF) chicken serum**. APEC strains were incubated with different dilutions of SPF chicken serum at 37 °C for 30 min and the surviving bacteria were counted. Mutant strain shows decreased survival in SPF chicken serum compared with wild-type and complemented strains. A two-way ANOVA was performed for the survival assays (*P < 0.05).

During infection, resistance to bactericidal serum is essential for the systemic dissemination and survival of APEC [33, 34]. Thus, the effect of *dctR* on the serum resistance of CE08 was determined. Our results show that the survival of mutant strain ΔdctR was significantly reduced in 100% and 50% specific-pathogen-free (SPF) chicken serum compared with that of the wild-type strain CE08 (*P* < 0.05). The serum resistance defects were rescued by *dctR* gene complementation. However, no differences were observed among the wild-type, mutant and complemented strains in the heat-inactivated serum (Figure  3), this is due to the lack of functional antibacterial factor, such as complement. These observations indicate that DctR could enhance serum resistance of CE08.

### *dctR* promoted the adhesion of CE08 to DF-1 cells

**Figure 4:**
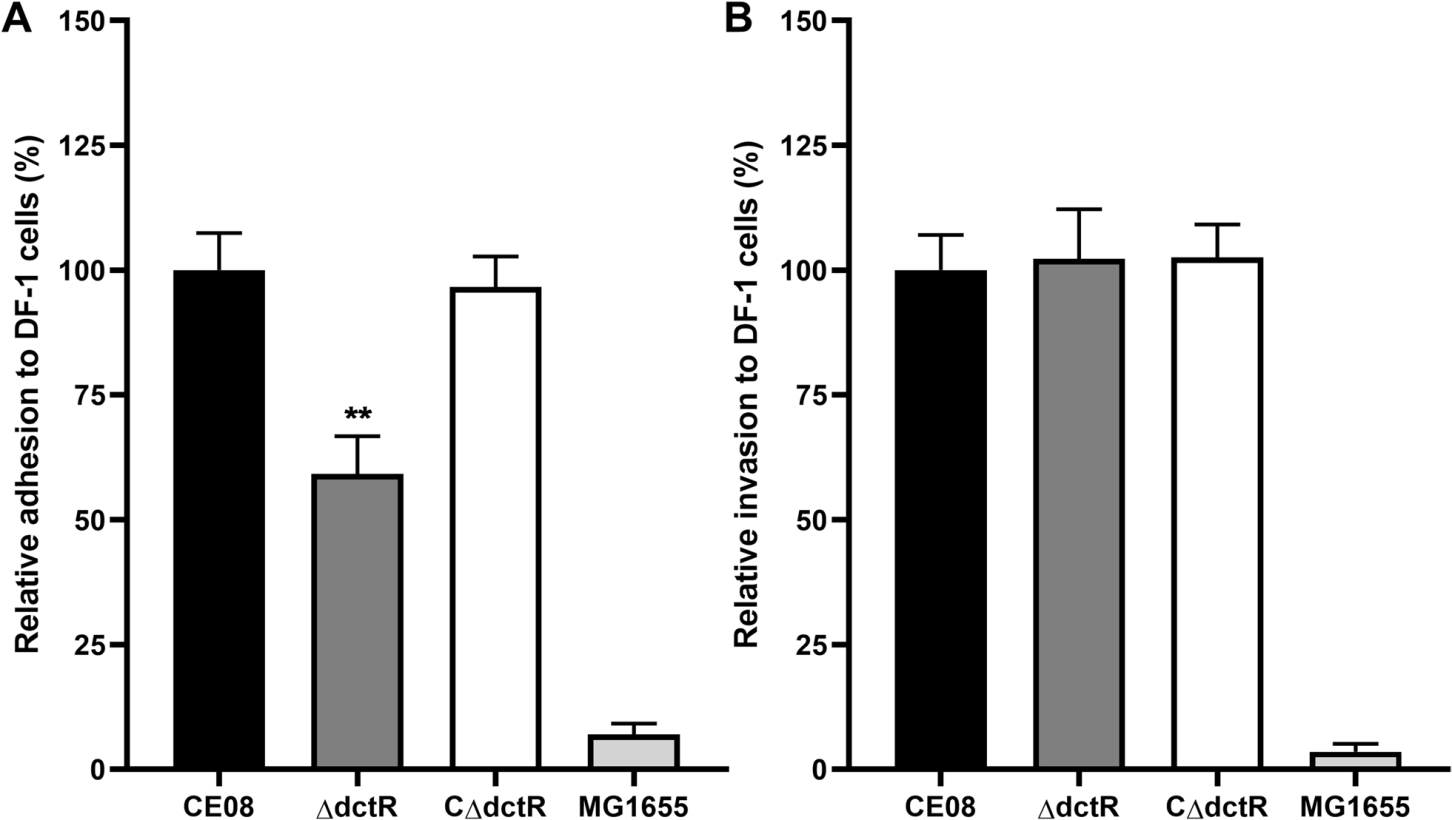
**Bacterial adherence and invasion assays**. The DF-1 cells were infected with APEC strains for 2 h and the adherent bacteria were counted. The invasion bacteria were determined by treatment with gentamicin to kill extracellular bacteria. The commensal *E*. *coli* K12 MG1655 was used as a negative control. **A** The mutant strain ΔdctR showed significantly decreased adhesion capacity compared with the wild-type and complemented strains. **B** No significant difference was observed for the invasion capacities among these APEC strains. Statistical significance was assessed using one-way ANOVA (**P < 0.01).

The capacities of adherence to and invasion into avian cell line DF-1 were compared among the wild-type, mutant and complemented strains to determine the roles of *dctR* involved in bacterial adhesion and invasion. The inactivation of the *dctR* gene led to significantly reduced adhesion to DF-1 cells by 41% compared to the wild-type strain CE08 (*P* < 0.01). The complemented strains restored the adhesion capacity. However, the invasion capacity of APEC was not affected by *dctR* deletion. Compared with the APEC, the commensal *E*. *coli* K12 MG1655 was unable to effectively adhere to and invade into DF-1 cells (Figure  4). The influence of *dctR* on the intracellular survival and replication of APEC in HD-11 chicken macrophages was also investigated. Although the mutant strain ΔdctR shows a slightly decreased intracellular survival, it was not significantly different from the wild-type and complemented strains at all time points tested (data not shown). These results indicate that *dctR* plays an important role in APEC adherence to DF-1 cells.

### *dctR* deletion attenuates CE08 virulence in vivo

**Figure 5:**
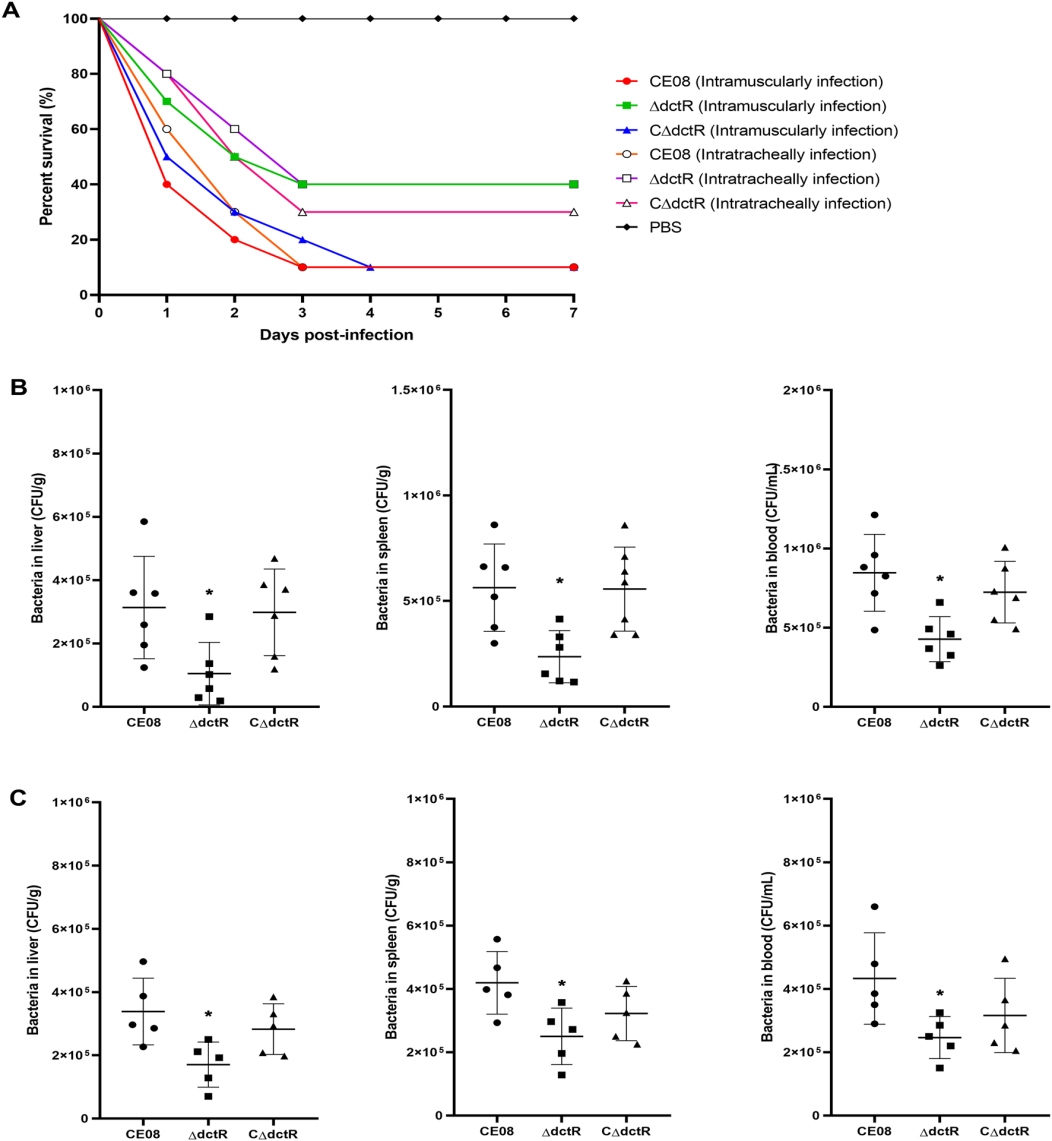
**DctR is required for the virulence and colonization of APEC during systemic infection**. **A** Determination of bacterial virulence. Seven-day-old ducks were infected with 10^6^ CFU of the APEC strains or negative control PBS. Survival was monitored for 7 days and the percent survival is shown. The virulence of the mutant strain was attenuated compared to the wild-type and complemented strains. **B**,** C** Colonization and survival of APEC strains in ducks during systemic infection. Seven-day-old ducks were infected intramuscularly (**B**) or intratracheally (**C**) with 10^8^ CFU of each APEC strain. Graphs show bacterial titers recovered from the liver, spleen and blood at 24 h post-infection. The bacterial loads of the mutant strain were significantly lower in liver, spleen and blood compared with the wild-type and complemented strains. A nonparametric Mann–Whitney U Test was conducted to determine statistical significance (*P < 0.05).

To investigate the effect of DctR on APEC pathogenicity, the duck infection models were used for virulence evaluation of wild-type, mutant and complemented strains. The survival curves indicate wild-type strain CE08 killed more ducks than the mutant strain ΔdctR. The mortality rates of ducks infected intramuscularly with CE08, ΔdctR, CΔdctR were 90% (9/10), 60% (6/10) and 90% (9/10), respectively. Therefore, the deletion of the *dctR* gene attenuated the virulence of APEC in ducks and the virulence could be restored in the complemented strain. A similar trend was observed for the intratracheal infection, although the complemented strain CΔdctR did not restore the virulence to the wild-type strain (Figure  5A). These results provide evidence that *dctR* is required for the full virulence of APEC.

### *dctR* is required for CE08 colonization in vivo

To address whether or not the *dctR* can affect the colonization and survival of APEC during systemic infection, the bacterial loads in the liver, spleen and blood were determined at 24 h post-infection. When inoculation was intramuscular, the amount of recovered mutant strain ΔdctR in the liver, spleen and blood was significantly reduced compared with the wild-type strain (*P* < 0.05). While the recovered complemented strain in tested organs and blood was restored to a level similar to that of the wild-type strain, it was significantly different from the mutant strain (*P* < 0.05) (Figure  5B). Similar results were also observed in the intratracheally infected duck (Figure  5C). These results indicate that DctR is required for effective colonization and survival of APEC during systemic infection in vivo.

### Inactivation of *dctR* downregulated the ETT2 and *traT* gene expression in CE08

**Figure 6:**
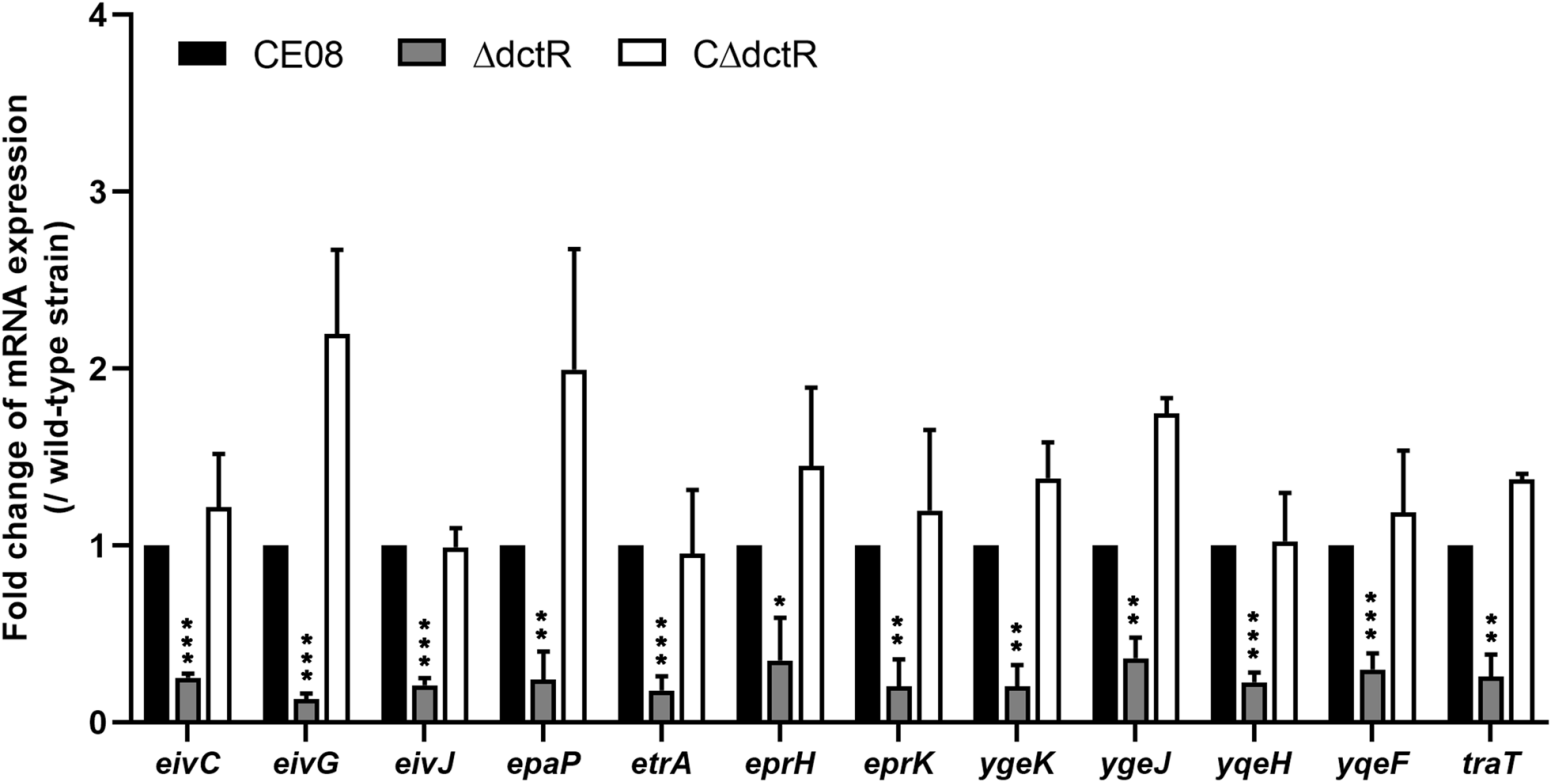
**Quantitative analysis of virulence gene transcription levels in APEC**. qRT-PCR was performed to measure the transcription levels of ETT2 core genes and virulence genes associated with serum resistance in wild-type, mutant and complemented strains. Transcription levels were normalized to the housekeeping gene *dnaE*. The results are shown as relative expression ratios compared to expression in the wild-type strain. Two-way ANOVA was carried out to determine statistical significance (*P < 0.05; **P < 0.01; ***P < 0.001).

Although the regulon of *dctR* is still unknown, it has been indicated that DctR negatively affects the expression of the LEE T3SS in EHEC O157:H7 [20, 21]. However, ExPEC strains, including APEC, do not contain a LEE locus. Thus, the transcription profiles of another T3SS, ETT2, in the APEC strains were analyzed by qRT-PCR. The transcriptions of ETT2 core genes *eivC*, *eivG*, *eivJ*, *epaP*, *etrA*, *eprH*, *eprK*, *ygeK*, *ygeJ*, *yqeH* and *yqeF* were significantly downregulated in the mutant strain ΔdctR, as compared to the wild-type strain (*P* < 0.05, *P* < 0.01, *P* < 0.001). Our results show that DctR contributes to the serum resistance of CE08, which prompted us to quantify the transcription levels of virulence genes associated with serum resistance. Deletion of the *dctR* gene resulted in significantly decreased transcription of *traT* gene (*P* < 0.01). While the expression of other serum resistance associated virulence genes, such as *ompA*, *iss*, were not influenced compared to the wild-type strain. The expression levels of ETT2 core genes and *traT* gene were restored in the complemented strain (Figure  6).

### Determination of inflammatory cytokine expression in APEC infected avian HD-11 cells

**Figure 7:**
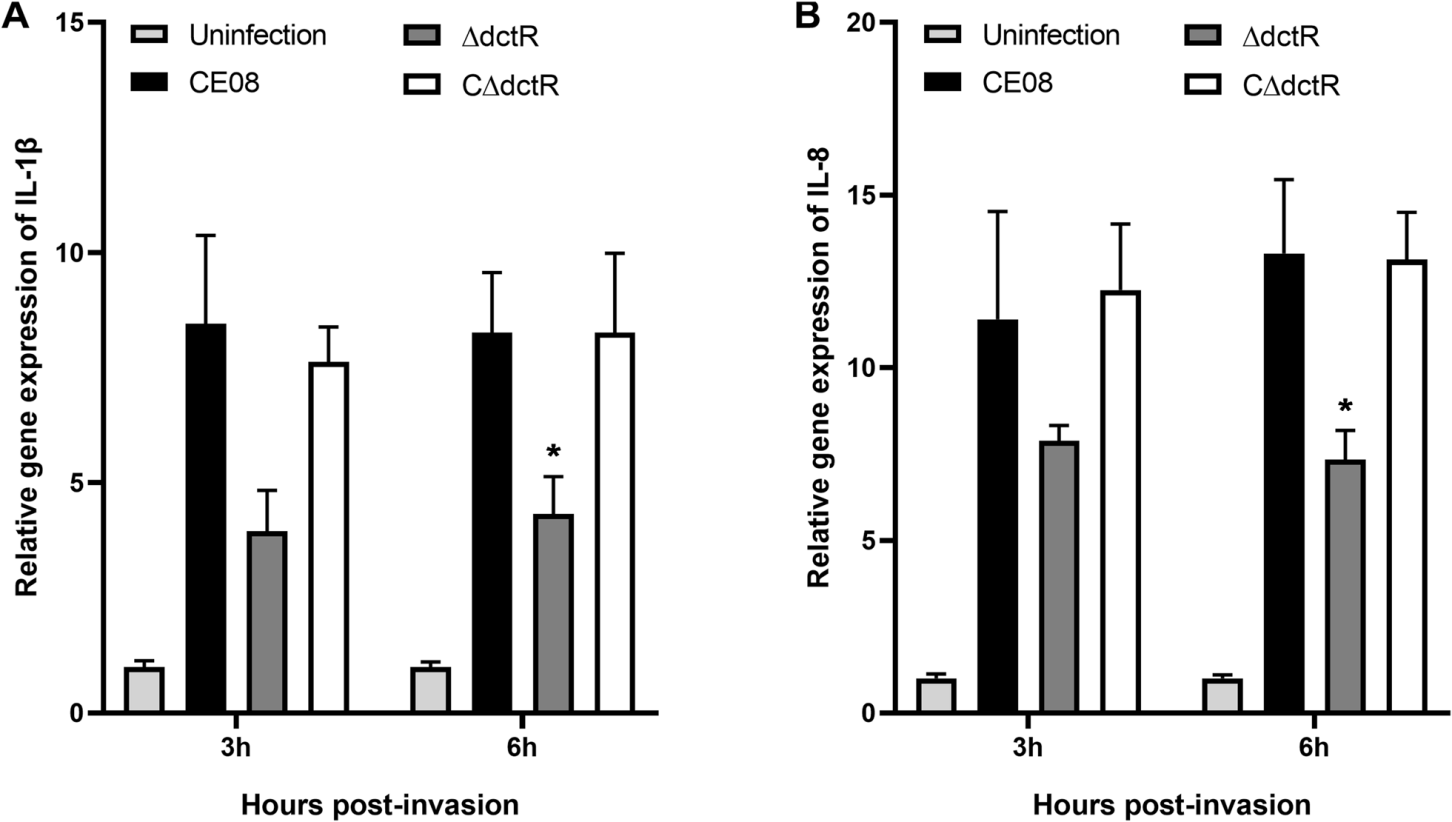
**Determination of inflammatory cytokine expressions in avian macrophages HD-11**. HD-11 cells were infected with each APEC strain. The transcription levels of IL-1β and IL-8 in bacteria-infected HD-11 cells were analyzed at 3 h, 6 h post-invasion by qRT-PCR. APEC infection led to significantly upregulated expressions of inflammatory cytokines IL-1β and IL-8 compared to the uninfected cells. The expression of IL-1β and IL-8 was significantly decreased in HD-11 cells infected with ΔdctR compared with the wild-type strain at 6 h post-invasion. Statistical significance was assessed by two-way ANOVA (*P < 0.05).

The pathogen infection could result in activation of macrophages and increases in mRNA expression of inflammatory cytokines [35]. Thus, we quantify the inflammatory cytokine expression of HD-11 cells infected with wild-type, mutant and complemented strains to investigate whether *dctR* gene deletion affect the host inflammatory responses. The expression of inflammatory cytokines IL-1β, IL-8 in infected HD-11 cells was analyzed at 3 h and 6 h post-invasion by qRT-PCR. As shown in Figure  7, the expressions of IL-1β and IL-8 were significantly upregulated in APEC infected HD-11 cells, relative to that of the uninfected cells. However, deletion of the *dctR* gene had obvious effects on the transcription of the inflammatory cytokines in APEC infected HD-11 macrophages. The expression of IL-1β and IL-8 were significantly downregulated in ΔdctR infected macrophages compared to that treated with wild-type (*P* < 0.05) at 6 h post-invasion. Moreover, the transcription of these inflammatory cytokines in CΔdctR infected macrophages was restored (Figure  7).

## Discussion

Bacterial pathogenesis requires exactly controlled expression of virulence factors, which could facilitate their adaptation and survival in the local microenvironment or host cells. In response to changing environmental cues, pathogenic bacteria rapidly coordinate the virulence gene expression through complex regulatory mechanisms, such as global transcription regulators, TCS, or the quorum sensing system [9]. LuxR family transcriptional regulators are widely present in most Gram-negative bacteria, which can alter the expression of a variety of genes, including those encoding virulence factors, antibiotic biosynthesis, motility, nodulation, plasmid transfer, bioluminescence and biofilm formation [12]. Moreover, various LuxR family transcriptional regulators have been characterized and involve the pathogenicity of *E*. *coli* [14–16]. Although DctR, a LuxR family regulator, has been demonstrated to participate in protection against metabolic end products under acidic conditions for *E*. *coli* [17, 18], the roles of *dctR* in APEC virulence remain unclear. Thus, we aimed at determining the effect of the DctR on the fitness and virulence of APEC. This study demonstrates that deletion of *dctR* significantly decreased the biofilm formation, colonization, survival fitness and virulence of APEC. In addition, we found that regulator DctR executed a positive regulation on ETT2 expression in APEC.

By employing a duck systemic infection model, we found that deletion of *dctR* markedly reduced the ability of APEC to effectively colonize and survive in vivo, subsequently attenuating virulence. These data suggest that DctR is required for the infection and virulence of APEC. Bacterial adherence and invasion to host cells play a critical role in the first step of pathogenesis by mediating colonization and infection. Various factors affect this process, such as fimbriae, adhesins, invasins, secretion systems, regulators [1, 36]. In this study, deletion of the DctR regulator significantly reduced the adhesion capacity of APEC to DF-1, but it did not affect the invasion capacity. The reduced capacity to adhere to DF-1 cells might contribute to the defects of the mutant strain to effectively colonize, survive in and infect ducks. However, increased adherent capacity to Caco-2 cells and bacterial shedding in feces were observed for the *dctR* mutant in EHEC O157:H7. This was due to DctR repressing the expression of LEE T3SS, whereas no regulation effect on other putative adherence-associated gene clusters is observed for the *dctR* mutant [20]. Although pathogenic *E*. *coli* share many virulence strategies, diverse *E*. *coli* pathovars, especially for intestinal pathogenic *E*. *coli* and ExPEC utilize different virulence factors and mechanisms to cause distinct clinical diseases. EHEC often colonizes the gut and the causative agent of severe gastroenteritis, while APEC initially infects poultry via the respiratory tract and lead to bacteremia and disease in bodily sites outside of the gastrointestinal tract [1]. The differences in local infection environments could prompt APEC and EHEC to modify the virulence gene expression by various regulatory mechanisms. This might be a reason for the opposite regulation effects on the adhesion of DctR in EHEC and APEC.

ExPEC, including APEC, is one of the leading causes of bloodstream infections, which spreads systemically through the entire body and results in extraintestinal diseases [3]. The complement system can mediate opsonization and bactericidal properties, thereby facilitating bacterial clearance during both localized and systemic infections. Thus, resistance to bactericidal serum is believed to play an important role in the pathogenicity of APEC strains. Moreover, it is indicated that serum resistance contributes to the septicemia and mortality of APEC [34, 37]. It is indicated that many virulence factors contribute to serum resistance of APEC, including OmpA, capsule, Increased serum survival (Iss) protein and transfer protein (TraT) [5, 33]. We demonstrate that the mutant strain was more susceptible to bactericidal serum, which might be explained by decreased expression of the *traT* gene due to *dctR* gene deletion. The reduced survival in the blood and virulence in vivo may be attributed to the serum resistance defects for the mutant strain. In addition, biofilm enables bacteria to overcome extreme environmental stresses, such as the host immune defenses and antibiotic treatment. Thus biofilm production contributes to the survival and enhanced virulence of pathogens [38]. In this study, regulator DctR also functioned in promoting biofilm formation of APEC. The RcsB, a response regulator of Rcs TCS, is involved in the control of various stress responses, motility and biofilm formation of *E*. *coli*. It is indicated that RcsB can form a heterodimer with *dctR* and modulate DNA binding specificity [23]. Therefore, the deletion of DctR may affect the function of RcsB and thus reduce the biofilm formation of APEC. The detailed mechanism of DctR affecting the biofilm formation of APEC remains to be determined in further studies. In addition, DctR provides protection against acid metabolite stress. Thus, the *dctR* mutant strain might be defective for countering the toxin effects of organic acids, which could be a reason for the decreased survival and virulence of the mutant strain in vivo.

LuxR family regulators usually contain a helix-turn-helix DNA binding domain for transcriptional regulation [12]. Previous studies have shown that DctR can repress the expression of the L-aspartic acid transporter gene *dctA* and LEE T3SS, then regulate the acid resistance and adhesion in EHEC O157:H7 [18, 21]. However, neither the DctR regulon is defined, nor direct evidence for the DctR binding sites has previously been presented. Unlike EHEC, ExPEC strains, including APEC, do not contain a LEE T3SS, but it harbors another T3SS, ETT2 [39, 40]. Our previous study verified that the ETT2 is wildly distributed in APEC, which might be a potential risk to human health [41]. Thus, the regulatory effect of DctR on ETT2 in APEC was determined. We found that the inactivation of DctR led to significant decreased expression of ETT2 core genes, suggesting that DctR positively regulated ETT2 in APEC. Moreover, ETT2 has been involved in bacterial surface properties, adhesion, serum resistance, survival and virulence of various pathogenic *E. coli*, including APEC [25, 26, 42, 43]. Thus, the downregulation of ETT2 might be responsible for the defects in adhesion, serum resistance, colonization and virulence of the mutant strain ΔdctR. Thus, our findings reinforce the importance of ETT2 in APEC virulence. Although the regulatory effects of DctR in APEC and EHEC O157 are opposite, the regulation of these two T3SS (LEE and ETT2) is consistent with their phenotype. Thus, this study provides evidence that DctR contributes to the virulence of APEC at least through regulation of ETT2 expression and function. Although we intend to investigate whether DctR bind to and directly regulate ETT2 expression, no putative DctR binding site was found in ETT2 according to the consensus sequence LuxR binding site reported previously [44], suggesting that DctR might indirectly regulate ETT2 expression. This possible regulation mechanism of DctR requires further study.

Once infected by a pathogen, the host macrophages could be activated and produce strong inflammatory responses in defense to pathogens. Nevertheless, pathogenic bacteria are able to efficiently subvert host inflammatory and immune responses, thereby facilitating their survival and infections [35, 45]. In the present study, we show that the mutant strain had a reduced capacity for induction of inflammatory cytokines, which might be a reason for the attenuated virulence because the excessive inflammation induced by APEC could lead to immune failure inflammatory damage. Bacteria utilize T3SS to deliver effector T3SS and regulate the inflammatory response [35]. Thus, APEC might interfere with the host inflammatory response by affecting the decreased ETT2 expression and its secretion function. However, the exact pathogenic mechanisms of ETT2 remain unknown, needing further investigation.

Although many virulence factors have been identified in APEC, the regulation of their expression is still not fully understood. Our study demonstrates that regulator DctR contributes to the adhesion, survival and colonization fitness as well as virulence of APEC. The most provocative finding of this study is that DctR functions on APEC virulence by regulating the expression and function of ETT2. However, the regulon and regulation mechanisms of DctR are unclear. Thus, further investigations are needed to determine the regulation effect breadth and molecular mechanisms of DctR, helping us to prevent poultry colibacillosis and potential human infections.

## Supplementary Information

**Additional file 1: Primers used in this study**.

## References

[1] Croxen MA, Finlay BB (2010). Molecular mechanisms of *Escherichia coli* pathogenicity. Nat Rev Microbiol.

[2] Kaper JB (2005). Pathogenic *Escherichia coli*. Int J Med Microbiol.

[3] Biran D, Ron EZ (2018). Extraintestinal Pathogenic *Escherichia coli*. Curr Top Microbiol Immunol.

[4] Guabiraba R, Schouler C (2015). Avian colibacillosis: still many black holes. FEMS Microbiol Lett.

[5] Ewers C, Li G, Wilking H, Kiessling S, Alt K, Antao EM, Laturnus C, Diehl I, Glodde S, Homeier T, Bohnke U, Steinruck H, Philipp HC, Wieler LH (2007). Avian pathogenic, uropathogenic, and newborn meningitis-causing *Escherichia coli*: how closely related are they?. Int J Med Microbiol.

[6] Tivendale KA, Logue CM, Kariyawasam S, Jordan D, Hussein A, Li G, Wannemuehler Y, Nolan LK (2010). Avian-pathogenic *Escherichia coli* strains are similar to neonatal meningitis *E*. *coli* strains and are able to cause meningitis in the rat model of human disease. Infect Immun.

[7] Belanger L, Garenaux A, Harel J, Boulianne M, Nadeau E, Dozois CM (2011). *Escherichia coli* from animal reservoirs as a potential source of human extraintestinal pathogenic *E*. *coli*. FEMS Immunol Med Microbiol.

[8] Mellata M (2013). Human and avian extraintestinal pathogenic *Escherichia coli*: infections, zoonotic risks, and antibiotic resistance trends. Foodborne Pathog Dis.

[9] Krell T, Lacal J, Busch A, Silva-Jimenez H, Guazzaroni ME, Ramos JL (2010). Bacterial sensor kinases: diversity in the recognition of environmental signals. Annu Rev Microbiol.

[10] Veening JW, Tamayo R (2018). Editorial overview: Bacterial cell regulation: from genes to complex environments. Curr Opin Microbiol.

[11] Hall CL, Lee VT (2018). Cyclic-di-GMP regulation of virulence in bacterial pathogens. Wiely Interdiscip Rev RNA.

[12] Chen J, Xie J (2011). Role and regulation of bacterial LuxR-like regulators. J Cell Biochem.

[13] Subramoni S, Florez Salcedo DV, Suarez-Moreno ZR (2015). A bioinformatic survey of distribution, conservation, and probable functions of LuxR solo regulators in bacteria. Front Cell Infect Microbiol.

[14] Lehti TA, Bauchart P, Dobrindt U, Korhonen TK, Westerlund-Wikstrom B (2012). The fimbriae activator MatA switches off motility in *Escherichia coli* by repression of the flagellar master operon *flhDC*. Microbiol.

[15] Navasa N, Ferrero MA, Rodriguez-Aparicio LB, Monteagudo-Mera A, Gutierrez S, Martinez-Blanco H (2019). The role of RcsA in the adaptation and survival of *Escherichia coli* K92. FEMS Microbiol Lett.

[16] Wiebe H, Gurlebeck D, Gross J, Dreck K, Pannen D, Ewers C, Wieler LH, Schnetz K (2015). YjjQ represses transcription of *flhDC* and additional loci in *Escherichia coli*. J Bacteriol.

[17] Mates AK, Sayed AK, Foster JW (2007). Products of the *Escherichia coli* acid fitness island attenuate metabolite stress at extremely low pH and mediate a cell density-dependent acid resistance. J Bacteriol.

[18] Boogerd FC, Boe L, Michelsen O, Jensen PR (1998). *atp* Mutants of *Escherichia coli* fail to grow on succinate due to a transport deficiency. J Bacteriol.

[19] Masuda N, Church GM (2003). Regulatory network of acid resistance genes in *Escherichia coli*. Mol Microbiol.

[20] Tatsuno I, Nagano K, Taguchi K, Rong L, Mori H, Sasakawa C (2003). Increased adherence to Caco-2 cells caused by disruption of the *yhiE* and *yhiF* genes in enterohemorrhagic *Escherichia coli* O157:H7. Infect Immun.

[21] Tree JJ, Roe AJ, Flockhart A, McAteer SP, Xu X, Shaw D, Mahajan A, Beatson SA, Best A, Lotz S, Woodward MJ, La Ragione R, Murphy KC, Leong JM, Gally DL (2011). Transcriptional regulators of the GAD acid stress island are carried by effector protein-encoding prophages and indirectly control type III secretion in enterohemorrhagic *Escherichia coli* O157:H7. Mol Microbiol.

[22] Slater SL, Sagfors AM, Pollard DJ, Ruano-Gallego D, Frankel G (2018). The type III secretion system of pathogenic *Escherichia coli*. Curr Top Microbiol Immunol.

[23] Pannen D, Fabisch M, Gausling L, Schnetz K (2016). Interaction of the RcsB Response regulator with auxiliary transcription regulators in *Escherichia coli*. J Biol Chem.

[24] Datsenko KA, Wanner BL (2000). One-step inactivation of chromosomal genes in *Escherichia coli* K-12 using PCR products. Proc Natl Acad Sci U S A.

[25] Wang S, Xu X, Liu X, Wang D, Liang H, Wu X, Tian M, Ding C, Wang G, Yu S (2017). *Escherichia coli* type III secretion system 2 regulator EtrA promotes virulence of avian pathogenic *Escherichia coli*. Microbiol.

[26] Wang S, Liu X, Xu X, Yang D, Wang D, Han X, Shi Y, Tian M, Ding C, Peng D, Yu S (2016). *Escherichia coli* type III secretion system 2 ATPase EivC is involved in the motility and virulence of avian pathogenic *Escherichia coli*. Front Microbiol.

[27] Wang Y, Yi L, Zhang Z, Fan H, Cheng X, Lu C (2014). Biofilm formation, host-cell adherence, and virulence genes regulation of *Streptococcus suis* in response to autoinducer-2 signaling. Curr Microbiol.

[28] Wang S, Niu C, Shi Z, Xia Y, Yaqoob M, Dai J, Lu C (2011). Effects of *ibeA* deletion on virulence and biofilm formation of avian pathogenic *Escherichia coli*. Infect Immun.

[29] Zuo J, Yin H, Hu J, Miao J, Chen Z, Qi K, Wang Z, Gong J, Phouthapane V, Jiang W, Mi R, Huang Y, Wang C, Han X (2019). Lsr operon is associated with AI-2 transfer and pathogenicity in avian pathogenic *Escherichia coli*. Vet Res.

[30] Yi Z, Wang D, Xin S, Zhou D, Li T, Tian M, Qi J, Ding C, Wang S, Yu S (2019). The CpxR regulates type VI secretion system 2 expression and facilitates the interbacterial competition activity and virulence of avian pathogenic *Escherichia coli*. Vet Res.

[31] Zhuge X, Sun Y, Jiang M, Wang J, Tang F, Xue F, Ren J, Zhu W, Dai J (2019). Acetate metabolic requirement of avian pathogenic *Escherichia coli* promotes its intracellular proliferation within macrophage. Vet Res.

[32] Livak KJ, Schmittgen TD (2001). Analysis of relative gene expression data using real-time quantitative PCR and the 2(-Delta Delta C(T)) Method. Methods.

[33] Miajlovic H, Smith SG (2014). Bacterial self-defence: how *Escherichia coli* evades serum killing. FEMS Microbiol Lett.

[34] Ma J, An C, Jiang F, Yao H, Logue C, Nolan LK, Li G (2018). Extraintestinal pathogenic *Escherichia coli* increase extracytoplasmic polysaccharide biosynthesis for serum resistance in response to bloodstream signals. Mol Microbiol.

[35] Raymond B, Young JC, Pallett M, Endres RG, Clements A, Frankel G (2013). Subversion of trafficking, apoptosis, and innate immunity by type III secretion system effectors. Trends Microbiol.

[36] Le Bouguenec C (2005). Adhesins and invasins of pathogenic *Escherichia coli*. Int J Med Microbiol.

[37] Mellata M, Dho-Moulin M, Dozois CM, Curtiss R, Brown PK, Arne P, Bree A, Desautels C, Fairbrother JM (2003). Role of virulence factors in resistance of avian pathogenic *Escherichia coli* to serum and in pathogenicity. Infect Immun.

[38] Campoccia D, Mirzaei R, Montanaro L, Arciola CR (2019). Hijacking of immune defences by biofilms: a multifront strategy. Biofouling.

[39] Ren CP, Chaudhuri RR, Fivian A, Bailey CM, Antonio M, Barnes WM, Pallen MJ (2004). The ETT2 gene cluster, encoding a second type III secretion system from *Escherichia coli*, is present in the majority of strains but has undergone widespread mutational attrition. J Bacteriol.

[40] Miyazaki J, Ba-Thein W, Kumao T, Akaza H, Hayashi H (2002). Identification of a type III secretion system in uropathogenic *Escherichia coli*. FEMS Microbiol Lett.

[41] Wang S, Liu X, Xu X, Zhao Y, Yang D, Han X, Tian M, Ding C, Peng D, Yu S (2016). *Escherichia coli* type III secretion system 2 (ETT2) is widely distributed in avian pathogenic *Escherichia coli* isolates from Eastern China. Epidemiol Infect.

[42] Shulman A, Yair Y, Biran D, Sura T, Otto A, Gophna U, Becher D, Hecker M, Ron EZ (2018). The *Escherichia coli* type III secretion system 2 has a global effect on cell surface. mBio.

[43] Yao Y, Xie Y, Perace D, Zhong Y, Lu J, Tao J, Guo X, Kim KS (2009). The type III secretion system is involved in the invasion and intracellular survival of *Escherichia coli* K1 in human brain microvascular endothelial cells. FEMS Microbiol Lett.

[44] Antunes LCM, Ferreira RBR, Lostroh CP, Greenberg EP (2008). A mutational analysis defines *Vibrio fischeri* LuxR binding sites. J Bacteriol.

[45] Figueira R, Holden DW (2012). Functions of the *Salmonella* pathogenicity island 2 (SPI-2) type III secretion system effectors. Microbiol.

