# Supplementary material for: DctR contributes to the virulence of avian pathogenic Escherichia coli through regulation of type III secretion system 2 expression
Source: Vet Res. 2021 Jul 6;52:101. doi: 10.1186/s13567-021-00970-6 (PMC8259166; doi:10.1186/s13567-021-00970-6)
Supplement: Supplementary file 1 — Additional file 1: Primers used in this study. [file 13567_2021_970_MOESM1_ESM.docx]

| **Primers** | **Sequence (5**′ **to 3**′**) ^a^** | **Target genes** | |
| --- | --- | --- | --- |
| **For gene deletion and complementation** | | |  |
| dctRup-F | GCAAAACGGATACGTTGTTAG | Upstream region of *dctR* | |
| dctRup-R | GAAGCAGCTCCAGCCTACACCATCGTATCCCTGGTAATTA | Upstream region of *dctR* | |
| pKD-F | TAATTACCAGGGATACGATGGTGTAGGCTGGAGCTGCTTC | pKD3 | |
| pKD-R | TCTTTATATTGTTCAATCGTTCCATATGAATATCCTCCTTAG | pKD3 | |
| dctRdown-F | CTAAGGAGGATATTCATATGGAACGATTGAACAATATAAAGA | Downstream region of *dctR* | |
| dctRdown-R | CTGGTGGCAGACGATTTG | Downstream region of *dctR* | |
| dctRin-F | AGAGATCGACGTAATGTTGC | *dctR* | |
| dctRin-R | ATTTGATTCGCAATGCAGCT | *dctR* | |
| dctRout-F | GGCAATAACCAACCTGATATTC | Upstream region of *dctR* | |
| dctRout-R | TGAGAATACGCCGCTTGAGTT | Downstream region of *dctR* | |
| dctRCo-F | TCCGAATTCATGTTTCTTATAATTACC | *dctR* | |
| dctRCo-R | GACCTCGAGCACCAGATAATTAATATGC | *dctR* | |
| **For RT-qPCR** | | |  |
| dnaE RT-F | ATGTCGGAGGCGTAAGGCT | *dnaE* | |
| dnaE RT-R | TCCAGGGCGTCAGTAAACAA | *dnaE* | |
| dctR RT-F | TCGACTTTGTCGCACTCATC | *dctR* | |
| dctR RT-R | TCGCAATGCAGCTCAGAA | *dctR* | |
| slp RT-F | CAGGTGTGGCATTTGAGAGA | *slp* | |
| slp RT-R | TCACCGCATTGGTGTAGTAAG | *slp* | |
| chuS RT-F | CATCAGGGCGATGCATTACTA | *chuS* | |
| chuS RT-R | TAACTCAAGCGGCGTATTCTC | *chuS* | |
| eivC RT-F | GAAACCAACGGGAGAGCAGT | *eivC* | |
| eivC RT-R | GATACGACGGTGGCAGTTCA | *eivC* | |
| eivG RT-F | TGTTGCTCGTCCTGTCGTTT | *eivG* | |
| eivG RT-R | TCCAATTCCGCAGTACGCTC | *eivG* | |
| eivJ RT-F | CGTGAAGCCCAACACAATGA | *eivJ* | |
| eivJ RT-R | TTTCTTGCTCCTCATACGGCT | *eivJ* | |
| epaP RT-F | CGTAATGATGCCGGTTGGGA | *epaP* | |
| epaP RT-R | AGCTAACCAACTCCGGTTCA | *epaP* | |
| etrA RT-F | TTACAGCGATTTGGTTGCGT | *etrA* | |
| etrA RT-R | TGCTCAAAAGGGTGTTTACAGG | *etrA* | |
| eprH RT-F | GGATGAAGCTGTCTGGGTCAA | *eprH* | |
| eprH RT-R | AGCATTCCGTTGCTTACTCAC | *eprH* | |
| eprK RT-F | ATCCTGCTGTTGTGCGGTT | *eprK* | |
| eprK RT-R | CGCAGAAGCAAAATCCGTTG | *eprK* | |
| ygeK RT-F | ATGAGGTTGTTGGGGGCTAT | *ygeK* | |
| ygeK RT-R | AGTTCGGACCCCATCATTCC | *ygeK* | |
| ygeJ RT-F | TAAACCAGATGCACATTCCCA | *ygeJ* | |
| ygeJ RT-R | AGACATTGCCACGTTACCCC | *ygeJ* | |
| yqeH RT-F | ACATGGGGCTTTGTAGTCGTT | *yqeH* | |
| yqeH RT-R | CCTTCCACCACACCACGATT | *yqeH* | |
| yqeF RT-F | TGCTTGAATGGGATGAGCGT | *yqeF* | |
| yqeF RT-R | TTGCGGGCATTACGTTTCAC | *yqeF* | |
| traT RT-F | CAGCAATCAAGAAGCGTAAC | *traT* | |
| traT RT-R | TTCGCCTGAATCCAGTAGTA | *traT* | |
| iss RT-F | CCGACAGCAGTAACACCAAAGG | *iss* | |
| iss RT-R | TTCTGCACCGCCAACAAATT | *iss* | |
| ompA RT-F | GCTGAGCCTGGGTGTTTCCT | *ompA* | |
| ompA RT-R | TCCAGAGCAGCCTGACCTTC | *ompA* | |
| chβactin-F | GAGAAATTGTGCGTGACATCA | *β-actin* | |
| chβactin-R | CCTGAACCTCTCATTGCCA | *β-actin* | |
| chIL1β-F | TGGGCATCAAGGGCTACA | *IL-1β* | |
| chIL1β-R | TCGGGTTGGTTGGTGATG | *IL-1β* | |
| chIL8-F | TTGGAAGCCACTTCAGTCAGAC | *IL-8* | |
| chIL8-R | GGAGCAGGAGGAATTACCAGTT | *IL-8* | |

^a^ Restriction sites are underlined
